# An Assessment of the Potential Use of BNNTs for Boron Neutron Capture Therapy

**DOI:** 10.3390/nano7040082

**Published:** 2017-04-12

**Authors:** Tiago H. Ferreira, Marcelo C. Miranda, Zildete Rocha, Alexandre S. Leal, Dawidson A. Gomes, Edesia M. B. Sousa

**Affiliations:** 1 Centro de Desenvolvimento da Tecnologia Nuclear (CDTN), Avenida Presidente Antônio Carlos, 6627 Pampulha, Belo Horizonte 31270-901, MG, Brazil; tiago.hilario@hotmail.com (T.H.F.); zildeter7@gmail.com (Z.R.); asleal@cdtn.br (A.S.L.); 2 Departamento de Bioquímica e Imunologia—ICB—UFMG, Avenida Presidente Antônio Carlos, 6627 Pampulha, Belo Horizonte 31270-901, MG, Brazil; marcelocdem@gmail.com (M.C.M.); dawidson.gomes@gmail.com (D.A.G.)

**Keywords:** boron nitride nanotubes, BNNTs, bioapplications, boron neutron capture therapy, BNCT, cancer therapy

## Abstract

Currently, nanostructured compounds have been standing out for their optical, mechanical, and chemical features and for the possibilities of manipulation and regulation of complex biological processes. One of these compounds is boron nitride nanotubes (BNNTs), which are a nanostructured material analog to carbon nanotubes, but formed of nitrogen and boron atoms. BNNTs present high thermal stability along with high chemical inertia. Among biological applications, its biocompatibility, cellular uptake, and functionalization potential can be highlighted, in addition to its eased utilization due to its nanometric size and tumor cell internalization. When it comes to new forms of therapy, we can draw attention to boron neutron capture therapy (BNCT), an experimental radiotherapy characterized by a boron-10 isotope carrier inside the target and a thermal neutron beam focused on it. The activation of the boron-10 atom by a neutron generates a lithium atom, a gamma ray, and an alpha particle, which can be used to destroy tumor tissues. The aim of this work was to use BNNTs as a boron-10 carrier for BNCT and to demonstrate its potential. The nanomaterial was characterized through XRD, FTIR, and SEM. The WST-8 assay was performed to confirm the cell viability of BNNTs. The cells treated with BNNTs were irradiated with the neutron beam of a Triga reactor, and the apoptosis caused by the activation of the BNNTs was measured with a calcein AM/propidium iodide test. The results demonstrate that this nanomaterial is a promising candidate for cancer therapy through BNCT.

## 1. Introduction

Currently, several research groups have been studying the use of nanostructured materials for biomedical applications. In particular, the intrinsic optical, magnetic, mechanical, and chemical properties of these nanomaterials offer new opportunities to manipulate and regulate complex biological processes. These features allow, for example, major advances in the fields of molecular biology and bioengineering through the release of specific molecules in a targeted way [1,2,3].

In this context, boron nitride nanotubes (BNNTs) can be highlighted. These are nanostructures with boron and nitrogen atoms interleaved, forming a hybrid resonance with high thermal stability and good chemical inertness [4,5]. Furthermore, BNNTs present a low probability of degrading or releasing their content at undesired sites prematurely or into the bloodstream, so they are safer and stable.

Recent studies have shown that BNNTs present no apparent toxicity in the concentrations required for their good performance [6]. It has been demonstrated that BNNTs are internalized by cells [7], can efficiently deliver DNA [8], and improve the process of gene transfection [9] and the differentiation of mesenchymal stem cells [10]. Furthermore, a theoretical study by Hilder and Hill shows that BNNTs are suitable for drug encapsulation [11].

**Figure 1:**
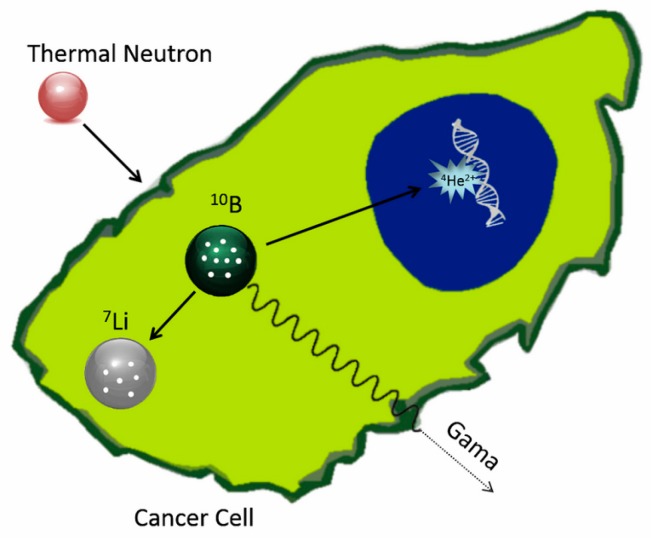
Schematic representation of activation of ^10^B through thermal neutron irradiation (Reprinted with permission from [16]; copyright (2016) Elsevier).

Boron neutron capture therapy (BNCT) is an experimental radiotherapy in which a compound containing atoms of the isotope ^10^B is administered to the patient and accumulated preferentially in tumor tissue. When ^10^B atoms contained in the tumor are irradiated with low energy neutrons, a spontaneous nuclear reaction occurs [12]. The neutrons are absorbed, and alpha radiation and lithium particles are produced within the tumor (Figure 1), destroying the cancer cells [13]. The BNCT associates a radiosensitizer (^10^B) with nonionizing neutron radiation, constituting a binary system. One of the main advantages of this type of system is the possibility of manipulating each component independently to improve its selectivity [14,15].

For BNCT to be successful, a sufficient number of ^10^B atoms (approximately 109 atoms/cell) must be delivered selectively to the tumor, and enough thermal neutrons must be absorbed by them to sustain a lethal ^10^B(n,a)^7^Li-capture reaction [13]. The main drawback of this therapy is related to the fact that the molecules commonly used have low amount of boron atoms. The use of BNNTs solves the current limitations of BNCT, since these nanotubes have large percentage of boron in its composition [17,18]. Furthermore, the irradiation of BNNTs, after they have been taken up by cells, increases the effectiveness of the therapy and reduces the effects on the surrounding healthy tissues [19]. This possibility would thus represent an actual magic bullet against tumors [20].

Tumor formation and growth are dependent on a gene modification that allows cells to be resistant to many stimuli that might trigger cell death and tumor control. To overcome this, some cancer treatments aim to stimulate various cell death pathways, such as programed cell death. BNNTs energy liberation during irradiation within cells can theoretically modify important structures, such as DNA and RNA, in a way that the tumor cell would not be able to repair. Those modifications can signal cell death induction. These cells may yet be submitted to such a large amount of energy released by the irradiated BNNT particles that it would lead to organelle and cell membrane disruption, exposing the cellular content to the environment, generating inflammation, and consequently stimulating the immune system [21,22,23,24]. 

In this study, BNNTs were irradiated in the nuclear reactor Triga (CDTN, Belo Horizonte, Brazil) with a thermal neutron flux, and the nuclear reaction was quantified by alpha detectors CR-39 (Landauer, IL, USA). The efficiency of BNCT in causing damage to the tumor cells using BNNTs was demonstrated through a cell death assay. It was demonstrated that nanotubes taken up by the cells cause more cell death than the non-internalized nanotubes, thereby improving the efficiency of this therapy.

## 2. Materials and Methods

Amorphous boron powder, ammonium nitrate, and hematite were obtained from Sigma-Aldrich (São Paulo, Brazil). All solvents used in this study were of analytical grade. All other chemicals used in this study were available commercially at a reagent grade and were used without further purification. MilliQ^®^ water (simplicity 185, Millipore, Bedford, MA, USA) was used throughout the study. 

### 2.1. Synthesis of BNNTs

BNNT samples were obtained from the synthesis based on the process described by Ferreira et al. [25]. The powders of NH_4_NO_3_ (95%, *w*/*w*), amorphous boron (97%, *w*/*w*), and hematite (95%, *w*/*w* and particle size less than 50 nm) were mixed at a weight ratio of 22:15:1, respectively, placed in tubular furnace and heat-treated. Then, the BNNTs were purified with hydrochloric acid solution (3 M) at 90 °C for 10 min, and the sample was collected by filtration afterwards and dried at 40 °C. Then, 50 mg of BNNTs were dispersed into 50 mL of HNO_3_ (65% *w*/*w*) and submitted to an ultrasound bath for 1 h. The dispersion obtained was transferred to a round bottom flask under reflux and stirred overnight at 60 °C. This step promotes the hydroxylation of the nanotubes (BNNT-OH) [26].

### 2.2. Physicochemical Characterization of BNNTs

The chemical and crystalline structure and the morphological features of BNNTs were characterized by Fourier transform infrared spectroscopy (FTIR), X-ray diffraction (XRD), and scanning electron microscopy (SEM). The FTIR analysis was carried out on a Thermo Nicolet 6700 spectrophotometer (Thermo Scientific, Waltham, MA, USA), ranging from 4000 to 600 cm^−1^ and with a resolution of 4 cm^−1^. The crystalline phases of samples were examined with X-ray powder diffraction. The XRD patterns were obtained using a Rigaku Geigerflex-3034 diffractometer (Rigaku, Tokio, Japan) with a Cu Kα tube. The SEM images were obtained through a field emission scanning electron microscope (FE-SEM) Sigma VP, Carl Zeiss (Carl Zeiss, Jena, Germany).

### 2.3. Quantification of ^10^B Activation

The solid state nuclear track detector (SSNTD) CR-39 (Landauer, IL, USA), with composition C_12_H_18_O_7_, is an promising alpha detector for BNCT purposes because, besides its ability to monitor sample activation, it can also be used as a tissue equivalent material for fast neutron microdosimetry, since it has almost the same composition as tissue [27,28].

CR-39 detectors were covered with 10, 50, and 100 μg of BNNTs. The samples were dispersed in MilliQ^®^ water and dropped onto the surface of detector. The CR-39 detectors were irradiated for 1 h in a TRIGA Mark-1 (nuclear research reactor of CDTN) with the power of 100 W and thermal neutron flux of 6.6 × 10^8^ n·cm^−2^·s^−1^. A detector without the presence of BNNTs was irradiated in the same conditions as a control.

After the irradiation, the CR-39 detectors were chemically etched with a 6.25 M solution of NaOH and 2% of alcohol at 75 °C for 14 h. The background was determined by processing the unexposed detector under identical conditions. The detectors were scanned using an optical microscope (ORTHOLUX, Leica Microsystems Ltd., Wetzlar, Germany) at a 5× objective lens coupled to a DFC295 camera (Leica Microsystems Ltd.). For each detector, 15 images were acquired using LASV3.8 software. The set of images was processed using a Quantikov Image Analyzer^®^ (developed at CDTN, Brazil). The track density per cm^2^ provides an estimate of the total number of tracks on the detector surface, which is proportional to the number of ^10^B atoms that were activated. 

### 2.4. Cytocompatibility Assays

#### 2.4.1. Cell Culture

In vitro assays were performed on HeLa cell line (ATCC CCL-2), derived from a human cervical adenocarcinoma. The HeLa cells were cultured at 37 °C in 5% CO_2_ in Dulbecco’s modified Eagle’s medium (Thermo Fisher Scientific, Waltham, MA, USA) containing 10% fetal bovine serum (FBS), 1 mM sodium pyruvate, 50 units/mL penicillin, and 100 μg/mL streptomycin (Thermo Fisher Scientific).

#### 2.4.2. Cell Viability Assay of the BNNTs

The WST-8 assay was used to evaluate cell viability, which are based on the conversion of a water-soluble tetrazolium salt, 2-(2-methoxy-4-nitrophenyl)-3-(4-nitrophenyl)-5-(2,4-disulfophenyl)-2H-tetrazolium, monosodium salt, into a water-soluble formazan dye upon reduction by dehydrogenases in the presence of an electron carrier [29]. HeLa cells (1 × 10^4^) were seeded onto a 96-well plate. The cells were then incubated with 0, 10, 50, 100, and 200 μg/mL of BNNTs. After 48 h of particles incubation, the extent of cell viability was assessed using the Cell Counting Kit-8 (CCK-8—Sigma-Aldrich). The CCK-8 solution was added to each well, followed by incubation for 2 h at 37 °C. The absorbance at 450 nm was determined using a microplate reader (Multiskan GO; Thermo Fisher Scientific). Cell viability was expressed as a percentage relative to the untreated cells (control). The experiments were carried out in triplicate. The significance of changes in treatment groups was determined by one-way analysis of variance and Bonferroni’s multiple comparison tests using Prism 6 software (La Jolla, CA, USA). Data were represented as mean ± S.E.M. 

### 2.5. Performance Test

In order to assess the efficiency of BNNTs as a system for BNCT, a new protocol was developed for the in vitro experiments.

#### 2.5.1. Groups Arrangement

1 × 10^5^ HeLa cells were resuspended in 1 mL of DMEM supplemented with HEPES buffer. The first group containing only cells was defined as the control group. The second group was treated with 100 μg/mL of BNNTs. Aiming to promote the internalization of the nanoparticles, HeLa cells were initially incubated with 20 μg/mL (20%) of BNNTs. After 12 h, these cells were washed with PBS, trypsinized, counted, and the same cell density were then incubated with the 80% remaining nanoparticles (80 μg/mL). This last group was defined as the third, BNNT uptake group.

#### 2.5.2. Cells Irradiation

The cells were irradiated for 1 h in the TRIGA Mark-1 nuclear research reactor of CDTN. Each sample contained 1 mL of HEPES/DMEM solution with 100 μg of BNNTs. During the irradiation, the reactor operated at the power of 100 W corresponding thermal neutron flux of 6.6 × 10^8^ n·cm^−2^·s^−1^. The control cell groups did not receive any irradiation.

#### 2.5.3. Cell Death Assay

After the procedure described in Section 2.5.2, 100 μL of each group were seeded onto a 96-well plate and supplemented with 1 μM calcein-AM (Thermo Fisher Scientific) and 2 μM propidium iodide (Life Technologies) in an HEPES/DMEM solution. Images were collected with a 4× objective lens on an Olympus IX70 inverted fluorescence microscopy (Olympus America, Melville, NY, USA). The data obtained were quantified by counting labeled cells using ImageJ software. Each treatment was made in triplicate, three different images were counted and the means were used for statistical analysis.

### 2.6. Statistical Analysis

The significance of changes in treatment groups was determined by one-way and two-way analyses of variance and Bonferroni’s multiple comparison test using Prism 6 software. Data are represented as the mean ± standard error of the mean (SEM).

## 3. Results and Discussion

### 3.1. BNNTs Characterization

**Figure 2:**
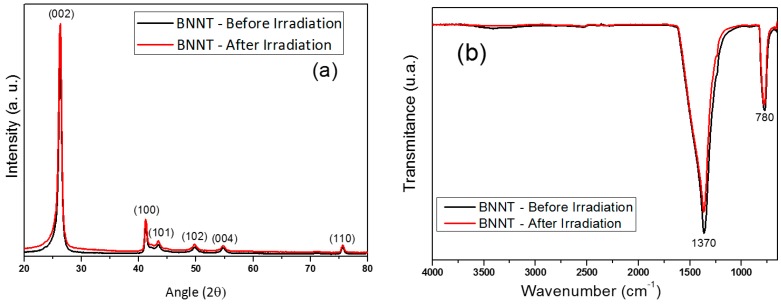
Diffraction patterns (**a**) and FTIR spectra (**b**) of boron nitride nanotubes (BNNTs) before and after irradiation.

The crystalline structures of the BNNTs before and after neutron irradiation were investigated by XRD; the profile is shown in Figure 2a. It is possible to observe the main peaks corresponding to the crystalline phase of the hexagonal boron nitride (hBN) at 2θ = 26.75°, 2θ = 41.58°, 2θ = 43.92°, 2θ = 50.16°, 2θ = 55.04° and 2θ = 75.86° (JCPDS, No. 9-12) corresponds to the hBN crystallographic planes (002), (100), (101), (102), (004), and (110), respectively. From the comparison between the two samples, it is not possible to notice significant changes in the crystalline structure of the material. This result shows that, even if neutron irradiation causes defects on the surface of the material, this irradiation is not sufficient to cause significant structural changes.

Figure 2b shows an FTIR spectrum of the BNNTs before (black) and after (red) irradiation. The most important feature displayed in the spectra is the strong asymmetric band centered at 1370 cm^−1^, corresponding to the B–N stretch bond, together with a less intense band at 780 cm^−1^ ascribed to the B–N–B bond [30]. Both spectra are quite similar, showing that there is no significant chemical change in the sample after irradiation with the neutron beam.

**Figure 3:**
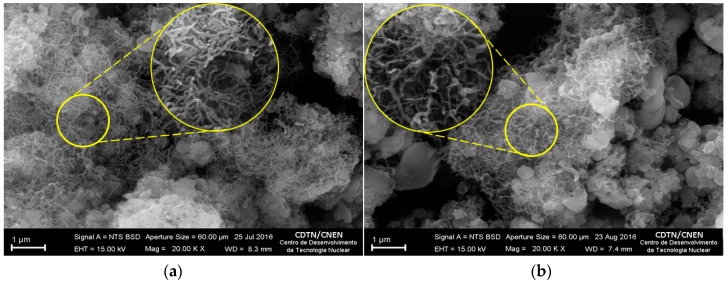
SEM images of BNNTs before (**a**) and after (**b**) irradiation.

Figure 3 shows representative SEM images of BNNTs before (Figure 3a) and after (Figure 3b) irradiation. Both images present a remarkable amount of nanotubes measuring between 50 and 70 nm in diameter and length up to 1 μm. Despite the presence of materials presenting a different shape of nanotubes in Figure 3b, this difference is not significant enough to be attributed to the irradiation process. In general, there was no significant difference in the morphology of nanotubes after irradiation. 

### 3.2. ^10^B(n,α)^7^Li Reaction Rate

The irradiation of the BNNTs with neutrons promotes the activation of ^10^B nuclides present in the sample according to the reaction: ^10^B(n,α)^7^Li. The production of ^7^Li and α particles is described by the reaction rate (*R*) defined in Equation (1).

*R* = φ_th_·N·σ·*m*. (1)

In this equation, the parameter σ is the cross section for the thermal neutrons of the reaction ^10^B(n,α)^7^Li. In this case, σ = 3830 barns (1 barn = 10^−24^ cm^2^). The parameter φ_th_ (6.6 × 10^8^ cm^2^·s^−1^) is the thermal neutron flux in the TRIGA IPR-R1 reactor in the position used for the irradiation. N is equivalent to 6.2 × 10^22^ atoms of ^10^B by unit of mass of just ^10^B and *m* is the mass (g) of just ^10^B dropped onto the surface of the detector. Considering these parameters, the value obtained for *R* is 1.52 × 10^11^ (^10^B nuclides) s^−1^. In this work, the samples were irradiated for 1 h, which means a total production of 9.2 × 10^−9^ alpha particles from the initial amount of the ^10^B target atoms. This result is in agreement with a previous work in which the activation of thin films of boron was studied [31].

Considering a mass of 100 μg, which is the amount of BNNTs used in some of the biological assays, 10% of this total consists of ^10^B atoms. On the other hand, the approximate number of alpha particles produced during irradiation is 2 × 10^8^. This amount of ^10^B is too small to provoke changes in the structure of the material, but it is enough to induce death in a large number of cells.

### 3.3. Quantification CR-39

**Figure 4:**
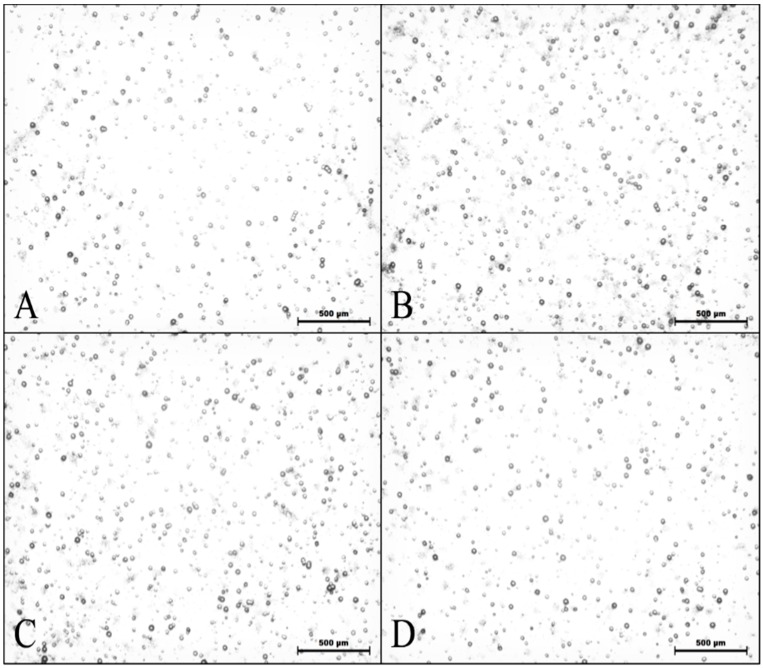
Images obtained by optical microscopy of solid traction detectors CR-39 irradiated with thermal neutrons for Samples (**A**) (10 μg of BNNTs), (**B**) (50 μg of BNNTs), (**C**) (100 μg BNNTs), and (**D**) (control).

The study of the detection of alpha particles released by neutron activation was performed after irradiation and development of CR-39 detectors. Figure 4 shows the images obtained by optical microscopy for samples containing 10, 50, and 100 μg of BNNTs. It is possible to observe tracks related to the alpha-detector interaction. Despite the presence of alpha particles produced by the activation process, the samples showed little difference between the tracks; dashes recorded on the pure detector sample are possibly from contamination.

Table 1 shows the number of tracks demonstrated and the quantity of tracks per unit of area assessed by the Quantikov Image Analyzer^®^ software from the optical microscopy images for the different samples. The track density per μm^2^ provides an estimate of the total number of tracks on the detector surface, which is proportional to the number of ^10^B atoms that were activated. 

A kind of calibration curve was established by using varying amounts of a well-known ^10^B material applied over the CR-39 surface before irradiation under reference flux. An alternative to the variation of material amount is the variation in irradiation time.

**Figure 5:**
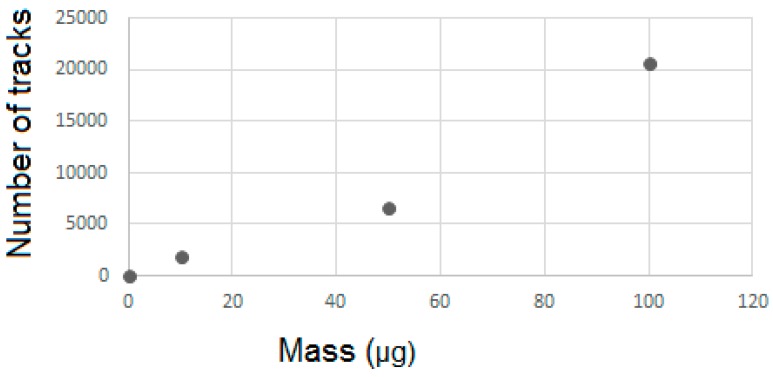
Number of effective tracks obtained by the optical microscopy study as function of BNNT content on the surface of the detector.

A number of effective tracks were obtained through the calibration of the data acquired from optical microscopy images. The results presented in Figure 5 show the real amount of tracks from the activated BNNTs, thus avoiding quantification of the background present in the reactor.

This analysis confirms that the results from the detection tests follow linear proportions, thus showing that the mass of nanomaterial deposited on the surface of the CR-39 detector is directly connected to the amount of tracks produced in its structure.

The CR-39 SSNTD efficiency is determined by the ratio between the number of tracks integrated over the surface of the CR-39 and the total number of ^10^B activated by neutron absorption. The total number of ^10^B atoms activated by neutron absorptions is determined in a reference thermal neutron flux integrated over the irradiation time. The value of the used reference flux is very stable and continuously monitored by activation detectors.

### 3.4. Cell Viability Assay of BNNT

**Figure 6:**
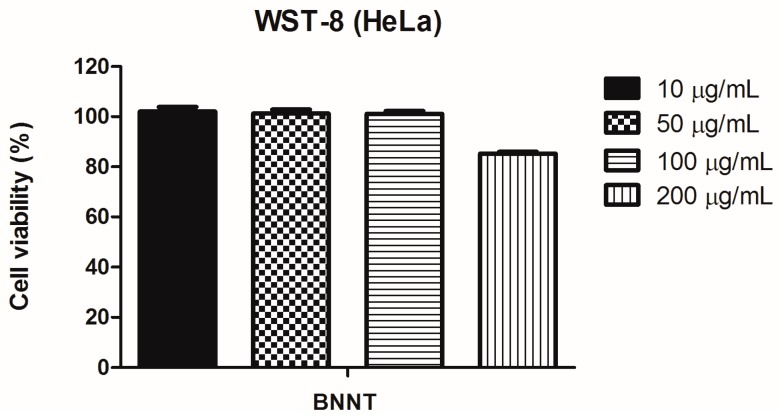
Cell viability of HeLa cells incubated with different concentrations of BNNTs (10, 50, 100, and 200 μg/mL). After 48 h, cells were incubated with CCK-8 solution for 2 h and the ability of attached cells to reduce WST-8 was calculated. The nanoparticle-treated groups were normalized in relation to cell viability of the control group (0 μg/mL).

Before irradiation of cells, the absence of cytotoxic effects has been verified. Figure 6 shows the quantitative results of WST-8 assay performed after 2 days of incubation with 0–200 μg/mL of BNNTs. The cell viability was more than 80% in all the studied concentrations, indicating no adverse effects on HeLa cultures in terms of metabolic activity after the incubation time. This result is in accordance with others studies that show the good biocompatibility of BNNTs up to 200 μg/mL [10,32,33].

### 3.5. Cell Irradiation Assay

**Figure 7:**
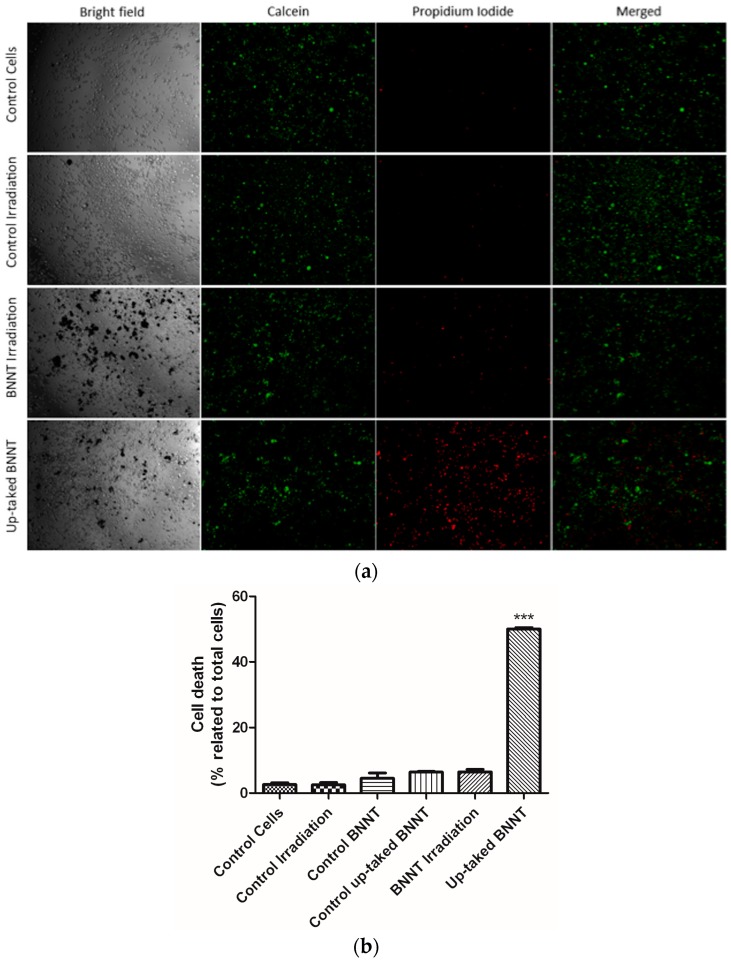
Cytotoxic effects of BNNTs on HeLa cells are higher in uptake cells and minor otherwise. (**a**) Live/Dead panel of control groups and irradiation treatment (live-green/dead-red). (**b**) Quantification of dead cells (% of total cells).

The efficacy of the BNNTs as a relevant agent for BNCT was evaluated from its capacity to promote cell death signaling in tumor cells through this treatment. Representative fluorescence microscopic panel of cells after 1 h of irradiation are presented in Figure 7a. Detection of a PI (DNA-binding red fluorophore) confirmed that cell death was significantly greater after irradiation in cells with BNNTs inside (50% ± 0.5) (Figure 7b). Otherwise, the control groups—cells, irradiation, BNNTs exposed, and uptake BNNTs without irradiation—exhibit high percentage of live cells (green)—97% ± 0.5, 98% ± 0.7, 96% ± 1.6, 96% ± 1.9, and 94% ± 0.8, respectively (Figure 7b). A small interference was also observed in cell viability after irradiation without any BNNTs internalized. Additionally, a minor toxicity effect was seen in the cells where BNNTs were internalized and were not irradiated, corroborating the WTS-8 results. This data suggests a relevant cytotoxic effect of internalized BNNTs, being particularly safe for cells that are not able to incorporate the nanoparticles.

## 4. Conclusions

The present study evaluated, in an innovative approach, the possible use of BNNTs as a tool for the treatment of cancer through BNCT. The results showed a large amount of nanotubes produced from the synthesis protocol used. Irradiation process does not cause changes in the structure of the material. The biological assays showed that BNNTs have a suitable cell viability and that irradiation with an appropriate flux of thermal neutrons do not cause significant damage in the cells studied. However, when it combined with the internalization of a large amount of boron, the irradiated BNNTs promote a significant amount of cell death. From these results, it is possible to conclude that BNNTs are a very important tool for boron neutron capture therapy.

## Figures and Tables

**Table 1:** Results from the software study of CR-39 images obtained by optical microscopy.

Samples	Number of Traces	Traces/μm^2^
10 μg (A)	13,396	1.710 × 10^−4^
50 μg (B)	18,226	2.314 × 10^−4^
100 μg (C)	32,201	4.046 × 10^−4^
Control (D)	11,577	1.432 × 10^−4^
